# The C-X-C Motif Chemokine Ligand 5, Which Exerts an Antioxidant Role by Inducing HO-1 Expression, Is C-X-C Motif Chemokine Receptor 2-Dependent in Human Prostate Stroma and Cancer Cells

**DOI:** 10.3390/antiox13121489

**Published:** 2024-12-05

**Authors:** Kang-Shuo Chang, Syue-Ting Chen, Shu-Yuan Hsu, Hsin-Ching Sung, Wei-Yin Lin, Ke-Hung Tsui, Yu-Hsiang Lin, Chen-Pang Hou, Horng-Heng Juang

**Affiliations:** 1 Department of Anatomy, College of Medicine, Chang Gung University, Kwei-Shan, Tao-Yuan 33302, Taiwan; d000016684@cgu.edu.tw (K.-S.C.); stchen2021@mail.cgu.edu.tw (S.-T.C.); hsusy@mail.cgu.edu.tw (S.-Y.H.); hcs@mail.cgu.edu.tw (H.-C.S.); 2 Department of Urology, Chang Gung Memorial Hospital-Linkou, Kwei-Shan, Tao-Yuan 33302, Taiwan; laserep@mail.cgu.edu.tw; 3 Graduate Institute of Biomedical Sciences, College of Medicine, Chang Gung University, Kwei-Shan, Tao-Yuan 33302, Taiwan; 4 Department of Internal Medicine, Chang Gung Memorial Hospital-Linkou, Kwei-Shan, Tao-Yuan 33302, Taiwan; stephany0908@cgmh.org.tw; 5 Department of Urology, Shuang Ho Hospital, New Taipei City 235041, Taiwan; t2130@s.tmu.edu.tw; 6 Department of Medicine, College of Medicine, Taipei Cancer Center, Taipei Medical University, Taipei 11031, Taiwan

**Keywords:** CXCL5, CXCR2, HO-1, ROS, antioxidant, myofibroblast, prostate cancer

## Abstract

While the C-X-C motif chemokine ligand 5 (CXCL5) is recognized as an inflammatory mediator and a potent attractant for immune cells, its functions within the human prostate remain unclear. This study explored the expression, functions, and regulatory mechanisms of CXCL5 in prostate stroma and cancer cells. CXCL5 secreted from prostate cancer cells enhanced neutrophil migration. CXCL5 induced cell proliferation and invasion of prostate cancer cells in vitro and tumorigenesis in a xenograft animal model. C-X-C motif chemokine receptor 2 (CXCR2) has been identified on the surface of prostate fibroblasts and cancer cells. The supernatant of LNCaP cells or CXCL5 overexpression enhanced the migration and contraction of prostate myofibroblast WPMY-1 cells; however, pretreatment with SB225002, a CXCR2 inhibitor, can reverse these effects. CXCL5 evinces antioxidant properties by upregulating heme oxygenase-1 (HO-1) to counteract H_2_O_2_-induced reactive oxygen species (ROS) in a CXCR2-dependent manner in WPMY-1 and prostate cancer cells. Our findings illustrate that CXCL5, through HO-1, plays a role in antioxidation, and determine that the CXCL5/CXCR2/HO-1 pathway facilitates antioxidative communication between fibroblasts and cancer cells in the prostate. Therefore, targeting the CXCL5/CXCR2 signaling pathway could provide a new strategy for managing oxidative stress within the prostate.

## 1. Introduction

CXCL5, also called epithelial neutrophil-activating protein 78 (ENA78), is a proangiogenic CXC-type chemokine, an inflammatory mediator, and a powerful attractant for immune cells [1]. Early studies found that CXCL5 is secreted not only by immune cells, such as monocytes, macrophages, and neutrophils, but also by epithelial cells, endothelial cells, and fibroblasts [2]. Since CXCL5 is a pro-inflammatory chemokine, CXCL5 functions to attach the neutrophil to tissues in early inflammation. Studies related to the prostate indicated that serum CXCL5 level is the relevant marker for men with benign prostatic hyperplasia (BPH), bacterial prostatitis, or chronic prostatitis, and a circulating marker involved in the inflammatory response [3,4,5]. However, other studies found that CXCL5 is overexpressed in various types of cancers, including prostate cancer, and is associated with tumor progression [6,7,8]. Reports revealed that CXCL5 expression is upregulated in castration-resistant prostate cancer (CRPC) and could be used as a serum marker and a target gene for CRPC and bone metastases [6,9,10]. Except for the well-known anti-inflammatory oncogene, there has been no report indicating the antioxidant characteristics of CXCL5 in prostate cancer; however, a recent study illustrated CXCL5 as having antioxidation characteristics in mouse adipocytes [11].

C-X-C chemokine receptor type 2 (CXCR2) is a member of the G protein-coupled receptor (GPCR) family and belongs to the chemokine receptor subfamily. CXCR2 activation by its ligands, including CXCL1-3 and CXCL5-8, triggers intracellular signaling pathways that influence several physiological processes, including leukocyte trafficking, angiogenesis, and inflammation [12]. CXCR2 is expressed in various cell types, such as neutrophils, monocytes, and certain cancer cells [13,14]; consequently, CXCR2 has become a potential therapeutic target for several cancers [15]. Recent studies have highlighted the role of the CXCR2 axis in prostate cancer metastasis [16]. In addition, the CXCL5/CXCR2 axis has been implicated in cancers, in which it can facilitate tumor progression and metastasis, including prostate cancer [17,18]. CXCR2 antagonists have been evaluated in preclinical and clinical studies to block the interaction between CXCR2 and its ligands, which can influence the interaction between cancer cells and the tumor microenvironment in prostate cancer [19,20,21].

Reactive oxygen species (ROS)-mediated oxidation stress has been associated with increased risk of prostate cancer. Prostate cancer cells adaptively upregulate antioxidant genes to detoxify from excessive ROS; furthermore, antioxidant treatment can encourage the proliferation of prostate epithelial cells early in prostate tumorigenesis [22,23]. Heme oxygenase-1 (HO-1) converts pro-oxidant heme to carbon monoxide, biliverdin, and iron to eliminate ROS, thus demonstrating an antioxidant effect and functioning as a tissue protector [24]. However, studies have implied that HO-1 plays a diverse role in tumor progression in various cancers, including prostate cancer [25]. Previous studies revealed that HO-1 attenuated ROS induced by H_2_O_2_ or pyocyanin treatment, thereby reducing cell apoptosis in prostate cancer cells in vitro, indicating that HO-1 has antioxidant properties in prostate cancer cells [26]. No reports have revealed the correlation between CXCL5 and HO-1, although CXCL5 has been reported to have antioxidant characteristics in adipocytes [11]. In this study, our objective is to elucidate the function of CXCL5 in human prostate cancer and stromal cells and to investigate the antioxidant characteristics of the CXCL5/CXCR2/HO-1 axis in human prostate cells.

## 2. Materials and Methods

### 2.1. Cell Culture and Chemicals

The human prostate PZ-HPV-7, CA-HPV-10, LNCaP, PC-3, and DU145 cell lines were obtained from the Bioresource Collection and Research Center (BCRC, Hsinchu, Taiwan) and cultured as previously described [27]. Human prostate stromal myofibroblast WPMY-1 cells were purchased from the American Type Culture Collection (ATCC; CRL-2854; Manassas, VA, USA). The cells were cultured as instructed by the manufacturers. RPMI-1640 medium was purchased from Life Technologies (Rockville, MD, USA), and fetal bovine serum was obtained from GE Healthcare Life Sciences HyClone Laboratories (South Logan, UT, USA). DAPI (4,6-diamino-2-phenylindole), propidium iodide, DCF-DA (2′,7′-dichlorofluorescin diacetate), H_2_O_2_, and SB225002 were purchased from Sigma-Aldrich Co. (St. Louis, MO, USA). H_2_O_2_ was dissolved in phosphate buffered saline (PBS) before use. According to the manufacturer’s instructions, other chemicals were dissolved in the suggested solvent.

### 2.2. CXCL5 Expression Vector and Transfection

Human CXCL5 cDNA in the pDNR-LIB vector (MGC:12304) was purchased from Invitrogen (Carlsbad, CA, USA). The human CXCL5 expression vector was constructed by cloning a full-length CXCL5 cDNA into the expression vector pcDNA3.1/zeo (Invitrogen) with the Bam HI and Hind III sites. Proper ligation was confirmed by extensive restriction mapping and sequencing. For stable transfection, LNCaP cells were electroporated (170 V, 80 ms pulse length, and one pulse setting) using an ECM 830 square wave electroporation system (BTX, San Diego, CA, USA). Transfected LNCaP (LN-CXCL5) cells were selected by 100 μg/mL of Zeocin (Invitrogen) for at least 4 generations. To construct mock-transfected LNCaP cells (LN-DNA), cells were transfected with a controlled expression vector and clonally selected in the same manner as the transfected cells. WPMY-1 cells were transiently transfected with CXCL5 expression vectors using the X-tremeGene HP DNA transfection reagent (Roche Diagnostics GmbH, Anaheim, Germany) for 48 h.

### 2.3. Gene Knockdown

Cells were seeded in 6-well plates for 1 day; then, the culture medium was replaced with RPMI-1640 medium plus 10% FCS and 5 μg/mL polybrene (Santa Cruz Biotechnology, Santa Cruz, CA, USA). Cells were transduced with CXCL5 shRNA or HMOX-1 lentiviral transduction particles (sc-39349-V and sc-44306-V, Santa Cruz Biotechnology). Two days after transduction, cells were selected by incubation with 10 μg/mL puromycin dihydrochloride (Cyrusbioscience, New Taipei City, Taiwan) for at least another 3 generations. Mock-transfected cells were transduced with control shRNA encoding a scrambled sequence and transduction particles (sc-108080, Santa Cruz Biotechnology), and clonally selected in the same manner as gene knockdown cells.

### 2.4. Neutrophil Migration Assay

The neutrophil migration assay was used to assess CXCL5-induced neutrophil migration. Neutrophils drawn from subjects’ peripheral blood were isolated by PolymorphPrep^TM^ (Axis-Shield PoC, Oslo, Norway) provided by Dr. I-Hsien Lin, Department of Microbiology and Immunology, Chang Gung University, Taiwan. Neutrophils (1.5 × 10^6^) were suspended in a 200 μL serum-free medium and added to the upper chamber of the transwell, with a pore size of 3.0 µm (PITP01250, Merck Millipore Ltd., Darmstadt, Germany). In addition, 500 μL of conditioned medium from mock-transfected LNCaP (LN-DNA), CXCL5-transfected LNCaP (LN-CXCL5), mocked knockdown PC-3 (PC_shCOL), or CXCL5 knockdown PC-3 (PC_shCXCL5) cells were added to the lower chamber of the 24-well plates and then incubated at 37 °C in a humidified atmosphere of 5% CO_2_ for 2.5 h. After incubation, the neutrophils were removed in the upper chamber and the insert was transferred to a clean-well-containing cell dissociation buffer (13151014, Thermo Fisher Scientific, Waltham, MA, USA) and maintained for another 30 min at 37 °C to dislodge the cells from the bottom side of the polycarbonate membrane. The total number of migratory cells, including dislodged and previously migratory cells from the lower well, were counted using an ADAM Cell Counter (Bulldog Bio Inc., Portsmouth, NH, USA).

### 2.5. Flow Cytometry Analysis of Surface Expression of CXCR2 in Prostate Cells

The flow cytometry analysis of cell immunofluorescent staining was used to determine the expression of CXCR2 in human prostate cells. Cells were cultured in a T25 flask and incubated for 2 days. After incubation, cells were harvested and fixed with 100% methanol for 15 min at room temperature. Cells were washed with PBS containing 1% bovine serum albumin (BSA) and incubated with PE-conjugated mouse anti-human CXCR2 antibody (FAB331P, R&D Systems Inc., Shanghai, China) or PE-conjugated mouse IgG2a control antibody (IC003P, R&D Systems Inc.) for 1 h at room temperature. After being washed three times with FACS buffer (PBS containing 1% BSA) to remove unbound antibodies, cells were analyzed by flow cytometry using an Attune NxT acoustic focusing cytometer and Attune^TM^ NxT software version 2.4 (Thermo Fisher Scientific Inc., Waltham, MA, USA).

### 2.6. Immunoblot Assay

Equal amounts of cell extracts, which were measured with a BCA protein assay kit, were separated on a 10% SDS-PAGE gel and then transferred and analyzed by the Western lightning plus-ECL detection system (Perkin Elmer, Inc., Waltham, MA, USA). Antibodies against heme oxygenase (HO-1; Hsp32, Stressgen, Victoria, BC, Canada), CXCL5 (ab126763, Abcam, Cambridge, MA, USA), α-SMA (ab5694, Abcam), CXCR2 (DF7095, Affinity Biosciences, Cincinnati, OH, USA), PSA (A0562, Dako Denmark A/S, Glostrup, Denmark), Fibronectin (#610078; BD Biosciences, Bedford, MA, USA), Cyclin A (C-19), Cyclin B1 (GNS1), Cyclin D1 (DCS-6), Cyclin E (M-20), Cdk2 (M2), Cdk4 (DCS-35), Cdk6 (B-10, Santa Cruz Biotechnology, Santa Cruz, CA, USA), E-cadherin (#610181, BD Biosciences), N-cadherin (GTX127345, GeneTex, Inc., Irvine, CA, USA), Snail (C15D3, Cell Signaling, Danvers, MA, USA), Slug (C19G7, Cell Signaling), Vimentin (AP2739B, Abgent, San Diego, CA, USA), and β-actin (T0022, Affinity Bioscience) were used. Protein bands were detected and quantified using the ChemiGenius image capture system (Syngene, Cambridge, UK) and ImageJ software (version 1.52a).

### 2.7. Real-Time Reverse Transcription–Polymerase Chain Reaction (RT-qPCR)

Total RNA from cells was isolated using a Trizol reagent. The cDNA was synthesized, and a real-time polymerase chain reaction (qPCR) was performed. The mRNA expressions of the genes were tested using TaqMan MGB probes labeled with FAM dye for human CXCL5 (Hs01099660_g1), HO-1 (Hs00157965_m1), and β-actin (Hs01060665_g1), which were purchased from Applied Biosystems (Foster City, CA, USA). Gene transcript levels were normalized to β-actin levels.

### 2.8. Enzyme-Linked Immunosorbent Assay

After incubation with 1 mL of RPMI 1640 medium with 10% FCS for 24 h, the conditioned media of PC_shCOL, PC_shCXCL5, LN-DNA, and LN-CXCL5 cells were collected for CXCL5 assays using a Quantikine ELISA human CXCL5/ENA-78 immunoassay kit, conducted as described by the manufacturer (Catalog #DX000; R&D Systems Inc.). After the cells were treated with drugs as indicated, the cell monolayers were washed twice with ice-cold PBS and suspended in 500 μL of PBS, the levels of PSA in the supernatants were measured, and the levels of PSA in each sample were adjusted by protein concentrations in the whole-cell extract, which were measured using a reagent kit of BCA protein assay, as described previously [27].

### 2.9. Cell Proliferation Assay

Cell proliferation after serum starvation for 24 h was measured using Ki67 assays, as previously described [28]. Briefly, cells were harvested and fixed with cold 70% ethanol overnight at −20 °C. Cells were stained with Ki67 antibody-conjugated PE (Ki67-PE; BD Pharmingen^TM^ PE Mouse Anti-Ki 67 Set 556027, BD Biosciences) for 30 min in the dark. The proportion of Ki67 positive cells was measured using an Attune NxT acoustic focusing cytometer (Thermo Fisher Scientific Inc., Waltham, MA, USA).

### 2.10. Cell Cycle Analysis

A total of 2 × 10^5^ cells were cultured in a T25 flask with RPMI 1640 containing 10% FBS and incubated for 24 h. Cells were serum-starved for 24 h and then the solution was replaced with 10% FCS RPMI-1640 medium for another 24 h. Cells were harvested and fixed in cold 70% ethanol at −20 °C overnight. Cells were centrifuged and washed with cold PBS and were then resuspended in 1 mL of PBS containing 0.1% Triton X-100, ribonuclease (0.5 mg/mL), and propidium iodide (0.05 mg/mL). The cell mixture was incubated at 4 °C overnight. Cell cycle analysis was performed using the Attune NxT acoustic focusing cytometer (Thermo Fisher Scientific Inc.).

### 2.11. Cell Contraction Assay

Cell contraction was performed using a cell contraction assay kit (CBA-201, Cell Biolabs, Inc., San Diego, CA, USA) as previously described [28]. Briefly, cells were collected and cell pellets were resuspended in RPMI medium at 2 × 10^5^ cells/100 μL. A total of 100 μL of the cell suspension was mixed with 400 μL of the cold Collagen Gel Working Solution, added to a 48-well plate, and incubated for 1 h at 37 °C to form a collagen lattice. After adding 0.5 mL of culture medium to the top of each collagen gel lattice, cell contraction was evaluated by comparing the percentage of collagen gel lattice at specified time points with the initial size of the collagen gel lattice at 0 h. The collagen gel lattice was photographed with Cytation 1 Cell Imaging MultiMode Reader (Aglinet BioTek Instruments, Inc., Santa Clara, CA, USA) at selected time points and measured using ImageJ software (version 1.52a).

### 2.12. Cell Migration

The cell migratory ability of WPMY-1 cells was determined using a wound healing assay, as previously described [28]. Cells were overgrown in 24-well plates; the monolayer cells were uniformly wounded with sterile pipette tips to make a straight scratch. The floated cells were cleared by washing with PBS and serum-free medium wad was added to the plate. Cell migration was evaluated by comparing the percentage of wound area at specified time points with the initial wound area at 0 h. The scratched area was photographed under the Cytation 1 Cell Imaging MultiMode Reader (Aglinet BioTek Instruments, Inc.) at selected time points and measured using BioTek Gen 5 software.

### 2.13. Cell Invasion Assay

The cell invasive assay used 24-well Matrigel invasion chambers with 8 µm diameter pore inserts (Millipore, Temecula, CA, USA), as previously described [27]. Following a 24 h incubation period, cells that migrated through the Matrigel-coated membrane were fixed with 4% paraformaldehyde and stained with 0.1% crystal violet solution for 30 min. The images were acquired using an inverted microscope (IX71, Olympus, Tokyo, Japan), and quantitative analysis was performed using ImageJ software (version 1.52a).

### 2.14. Reporter Vector Constructs and Reporter Assay

The reporter vector containing the enhancer/promoter (−41 to −5874) of the PSA gene was constructed [29]. The synthesized DNA fragment was cloned into the pGL3-Basic vector at the *Bgl II* cutting site. The DNA fragment containing the enhancer/promoter of the HO-1 gene (−4433 to +22) was subtracted from a BAC clone (CTA-286B10; Invitrogen) and cloned into the pGL3-Basic vector (Promega BioScience, San Luis Obispo, CA, USA) with the *Kpn I* and *Xma I* cutting sites as previously described [26]. Cells were seeded in 24-well plates 1 day before transfection and then transiently transfected with a luciferase reporter vector for an additional 48 h. Relative luciferase activities were measured in relative light units (RLU) and adjusted by protein concentrations as previously described [30].

### 2.15. ROS Detection with Flow Cytometry

ROS was analyzed as previously described [31]. Briefly, cells were trypsinized, washed, and suspended in RPMI medium with 10% FBS and 20 μM carboxy-H2DCFDA. Cells were then treated with or without 500 μM H_2_O_2_ for 30 min at 37 °C. The intensity of DCF-DA fluorescence was quantified using an Attune NxT acoustic focusing cytometer (Thermo Fisher Scientific Inc., Waltham, MA, USA).

### 2.16. Animal Model of Xenograft

These studies were carried out with the approval of the Chang Gung University Animal Research Committee (Permit Number: CGU109-148). In all procedures, every effort was made to minimize both the suffering of laboratory animals and the number of animals used, as previously described [28]. Male nude mice (BALB/cAnN-Foxn1) were purchased from the Animal Center of the National Science & Technology Council, Taiwan. Cells were detached with Gibco Versene solution (Life Technologies, Grand Island, NY, USA) and washed with RPMI1640 medium with 10% FCS, resuspended in a PBS solution, 6.5 × 10^6^ cell/100 μL, and injected into the lateral back wall close to the shoulder of each mouse. Growth xenograft measurements were taken using a vernier caliper every 2–3 days. Tumor volume was determined using the formula Volume = π/6 × larger diameter × (smaller diameter)^2^.

### 2.17. Statistical Analysis

Statistical analyses were performed using the SigmaStat program for Windows, version 2.03 (SPSS Inc., Chicago, IL, USA). Data were expressed as mean ± SE. Student’s *t*-test or one-way ANOVA was used to determine the significance of the difference with a *p*-value less than 0.05 (* *p* < 0.05) or 0.01 (** *p* < 0.01). Post hoc analysis was used to correct for multiple comparisons.

## 3. Results

### 3.1. Expressions of CXCL5 and CXCR2 in Prostate Cells

**Figure 1:**
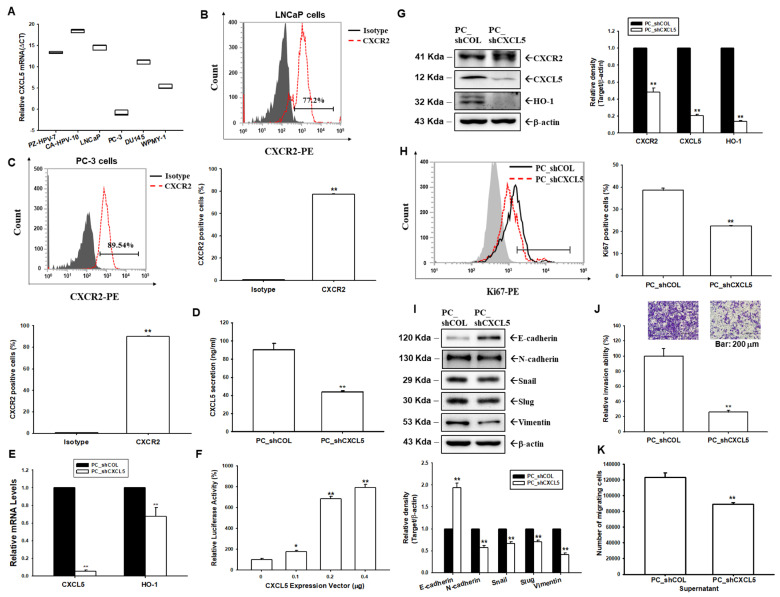
Modulation of CXCL5 in the cell proliferation and invasion of prostate cancer cells. (**A**) CXCL5 mRNA levels of prostate cells were determined by RT-qPCR. Data are presented as the ΔCT relative to β-actin. The cell immunofluorescent staining of CXCR2 protein in LNCaP (**B**) and PC-3 (**C**) cells was determined by flow cytometry. Data from quantitative analysis represented the percentage of CXCR2-positive cells. (**D**) CXCL5 levels in the supernatant from PC_shCOL and PC_shCXCL5 cells were assessed by ELISA. (**E**) The mRNA levels (±SE, *n* = 3) of CXCL5 and HO-1 of PC_shCOL cells relative to PC_shCXCL5 cells. (**F**) Reporter activity (±SE, *n* = 6) of HO-1 reporter vector after co-transfected with various dosages of CXCL5 expression vectors. (**G**) The protein levels of the CXCR2, CXCL5, and HO-1 of mock-transducted PC-3 (PC_shCOL) and CXCL5 knockdown PC-3 (PC_shCXCL5) cells were examined by immunoblot assays. Quantitative analysis (±SE, *n* = 3) was presented as a relative density of proteins/β-actin. (**H**) The abilities of cellular proliferation in PC_shCOL and PC_shCXCL5 cells were measured by flow cytometry using a Ki67 flow cytometry kit (±SE, *n* = 3). (**I**) The protein levels of EMT markers (E-cadherin, N-cadherin, Snail, Slug, and Vimentin) in PC_shCOL and PC_shCXCL5 cells were determined by immunoblot assays and quantitative analysis (±SE, *n* = 3). (**J**) The cellular invasion ability was determined by in vitro Matrigel invasion assays. Data are presented as the mean percentage (±SE; *n* = 3) in relation to the PC_shCOL cells. (**K**) The cell numbers for neutrophil trans-membrane migration induced by supernatant from PC_shCOL and PC_shCXCL5 cells (±SE, *n* = 3). * *p* < 0.05, ** *p* < 0.01.

The levels of CXCL5 mRNA in cultured prostate cells (PZ-HPV-7, CA-HPV-10, LNCaP, PC-3, DU145, and WPMY-1) were evaluated using RT-qPCR assays. The results showed that PC-3 cells express the highest levels of CXCL5 mRNA among the cells tested. Prostate stromal myofibroblast cells, WPMY-1, also express detectable levels of CXCL5 mRNA; however, PZ-HPV-7, CA-HPV-10, LNCaP, and DU145 cells express extremely low levels of CXCL5 mRNA (Figure 1A). The flow cytometry analysis of the immunofluorescent cell staining results confirmed that LNCaP cells (Figure 1B) and PC-3 cells (Figure 1C) express CXCR2.

### 3.2. Knockdown of CXCL5 Attenuates Cell Proliferation and Invasion Ability of PC-3 Cells

We constructed a stable CXCL5 knockdown PC-3 cell line by transducing lentiviral transduction particles of CXCL5 shRNA into PC-3 cells. ELISA (Figure 1D) assays confirmed that CXCL5 was knocked down in PC_shCXCL5 cells compared to mock knockdown PC cells (PC_shCOL). RT-qPCR assays showed that knockdown CXCL5 decreased HO-1 mRNA levels (Figure 1E). Further reporter assays indicated that transient overexpression of the CXCL5 expression vector increased the reporter activity of the HO-1 reporter vector (Figure 1F). Similar results were also found in immunoblot assays which showed that knockdown of CXCL5 downregulated CXCR2 and decreased HO-1 expression (Figure 1G). Ki67 proliferation assays revealed that PC_shCXCL5 cells have a lower proliferation ability than PC_shCOL cells (Figure 1H). Further immunoblot assays showed that CXCL5 knockdown enhanced E-cadherin but negatively regulated the expression of N-cadherin, Snail, Slug, and Vimentin (Figure 1I), which reduced PC-3 cell invasion ability (Figure 1J). The supernatant of PC_shCXCL5 cells negatively regulated the induction of neutrophil migration compared to the supernatant of PC_shCOL cells (Figure 1K).

### 3.3. Knockdown of CXCL5 Blocks Tumor Growth of PC-3 Cells in Animal Model

**Figure 2:**
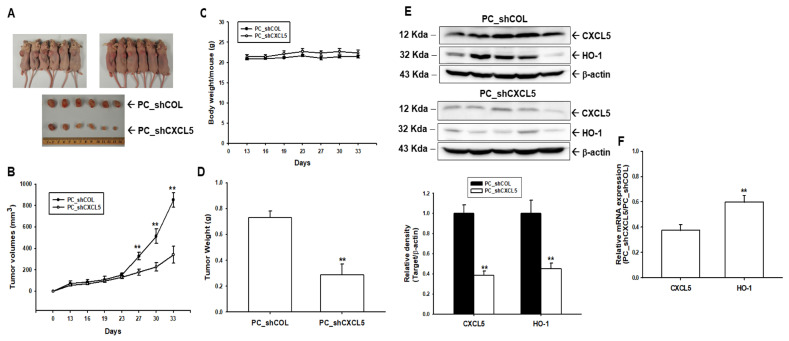
Knockdown of CXCL5 blocks PC-3 cell tumor growth of cells in xenograft mouse models. Athymic male nude mice were subcutaneously injected with PC_shCOL or PC_shCXCL5 cells for 33 days. (**A**) Photographs of representative xenografted mice and tumors. (**B**) The tumor sizes derived from PC_shCOL and PC_shCXCL5 were measured every 3 days. (**C**) Average body weights (mean ± SE) of mice during the experimental period. (**D**) Quantitative data (mean ± SE; *n* = 6) describing tumor weight of the PC_shCOL and PC_shCXCL5 groups when the mice were sacrificed on day 33. (**E**) Whole-cell lysates of tumor samples from the PC_shCOL and PC_shCXCL5 groups were subjected to immunoblot assays for CXCL5, HO-1, and β-actin. (**F**) The mRNA levels of CXCL5 and HO-1 in the xenografted tumors were analyzed using RT-qPCR assays (±SE, *n* = 3). ** *p* < 0.01.

We continued the research by seeking to determine whether CXCL5 knockdown affects tumor growth in xenograft studies. PC_shCOL and PC_shCXCL5 cells were xenografted into male nude mice (BALB/cAnN-Foxn1) for 33 days. The mice were sacrificed and tumor measurements recorded following the protocol approved by the Chang Gung University Animal Research Committee (Figure 2A). The xenograft study showed that, after inoculation, tumors in the PC_shCOL group grew considerably faster than those in the PC_shCXCL5 group. The final tumor volume was 853.50 ± 68.05 mm^3^ for the PC_shCOL group and 340.86 ± 78.61 mm^3^ for the PC_shCXCL5 group (Figure 2B). The average body weight between the PC_shCOL and PC_shCXCL5 groups was not significantly different during the inoculation period (Figure 2C). The average tumor weight was significantly higher in the PC_shCOL group compared to the PC_shCXCL5 group (0.73 ± 0.05 vs. 0.29 ± 0.08 g; Figure 2D). Immunoblot assays and quantitative analysis showed that CXCL5 knockdown negatively regulated HO-1 expression (Figure 2E) in xenograft tissues of the PC_shCXCL5 group, compared to the PC_shCOL group. The results of the RT-qPCR assay were similar (Figure 2F).

### 3.4. Knockdown of CXCL5 Enhances H_2_O_2_-Induced ROS in PC-3 Cells

**Figure 3:**
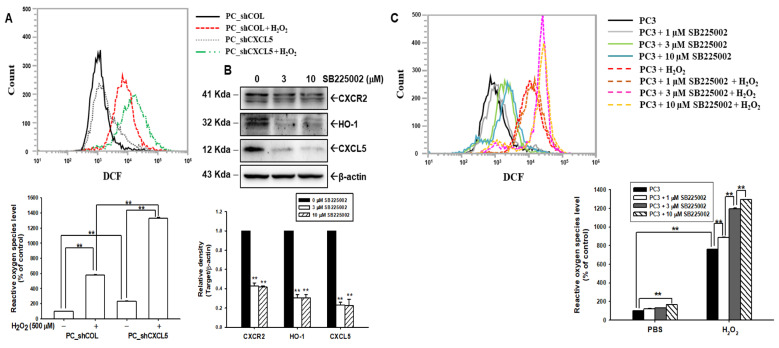
Modulation of CXCL5 and SB225002 in endogenous and H_2_O_2_-induced ROS in prostate cancer PC-3 cells. (**A**) ROS levels and quantitative data from PC_shCOL and PC_shCXCL5 cells after treatment with or without H_2_O_2_, as measured by flow cytometry. (**B**) The levels of the CXCR2, HO-1, and CXCL5 proteins after treatment with various concentrations of SB225002, as indicated, as examined by immunoblotting assays. Quantitative data are presented as the intensity of the protein bands of the target proteins/β-actin relative to the vehicle-treated group. (**C**) ROS levels and quantitative data from PC3 cells after treatment with various concentrations of SB225002 and with/without H_2_O_2_, as measured by flow cytometry. ** *p* < 0.01.

Since in vitro cell models and animal studies have found that CXCL5 blocked HO-1 expression, we continued on to determine whether CXCL5 has antioxidant capacity in human prostate cancer cells. The flow cytometry assays indicated that the knockdown of CXCL5 in PC-3 cells (PC_shCXCL5) induced a 2.31-fold increase in endogenous ROS, compared to PC_shCOL cells. Further flow cytometry assays and quantitative analysis showed that 500 µM H_2_O_2_ treatment induced 5.82-fold and 13.2-fold increases in ROS in PC_shCOL and PC_shCXCL5 cells, indicating that CXCL5 knockdown not only upregulated endogenous ROS but also H_2_O_2_-induced ROS in PC-3 cells (Figure 3A). Immunoblot assays revealed that CXCR2 inhibitor SB225002 treatments blocked CXCR2, CXCL5, and HO-1 expressions in PC-3 cells (Figure 3B). The flow cytometry assays and the quantitative analysis indicated that SB225002 treatments enhanced endogenous ROS and H_2_O_2_-induced ROS in PC-3 cells (Figure 3C), indicating that CXCL5 affects endogenous ROS and that H_2_O_2_-induced ROS is CXCR2-dependent.

### 3.5. Overexpression of CXCL5 Enhances Cell Proliferation Through Modulation of the Cell Cycle in LNCaP Cells

**Figure 4:**
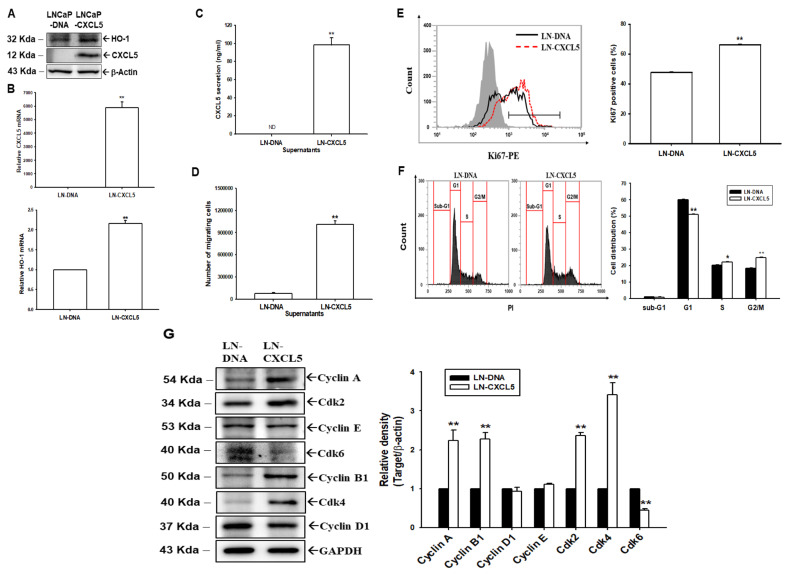
Modulation of CXCL5 in neutrophil migration and cell proliferation in prostate cancer LNCaP cells. (**A**) Protein levels of CXCL5 and HO-1 from mock-transfected LNCaP (LN-DNA) and LNCaP-overexpressed CXCL5 (LN-CXCL5) cells were examined by immunoblot assays. Quantitative analysis (±SE, *n* = 3) is presented as the relative density of target proteins/β-actin. (**B**) The mRNA levels (±SE, *n* = 3) of CXCL5 and the HO-1 of LN-DNA and LN-CXCL5 cells, as determined by RT-qPCR. (**C**) CXCL5 levels in the supernatant of LN-DNA and LN-CXCL5 cells, as evaluated by ELISA. (**D**) The numbers of neutrophil transmembrane migration cells induced by supernatant from LN-DNA and LN-CXCL5 cells (±SE, *n* = 3). (**E**) The cellular proliferation abilities of LN-CXCL5 relative to LN-DNA cells were measured by flow cytometry using a Ki67 flow cytometry kit (±SE, *n* = 3). (**F**) Cell cycle analysis of LN-DNA and LN-CXCL5 cells. (**G**) Cell cycle modulators’ protein levels were determined by immunoblot assays and quantitative analysis (±SE, *n* = 3). * *p* < 0.05, ** *p* < 0.01. ND: not detectable.

To support our concept that CXCL5 is an oncogene in human prostate cancer cells, we constructed a CXCL5-overexpressed LNCaP cell line. Immunoblot (Figure 4A), RT-qPCR (Figure 4B), and ELISA (Figure 4C) assays confirmed that CXCL5 overexpression in LNCaP cells (LN-CXCL5) increased CXCL5 and HO-1 expressions, compared to mock-transfected LNCaP cells (LN-DNA). The supernatant of LN-CXCL5 cells enhanced neutrophil migration compared to the supernatant of LN-DNA cells (Figure 4D). Ki67 proliferation assays revealed that LN-CXCL5 cells have a higher proliferation ability than LN-DNA cells (Figure 4E). Further cell cycle analysis showed that CXCL5 overexpression decreased the arrest of G1 but increased the population of G2/M cells (Figure 4F). Immunoblot assays revealed that overexpression of CXCL5 did not affect cyclin D1 and cyclin E expression but upregulated cyclin A, cyclin B1, Cdk2, and Cdk4. The levels of Cdk6 protein were blocked by overexpression of CXCL5 in LNCaP cells (Figure 4G).

### 3.6. Overexpression of CXCL5 Induces HO-1 and PSA in a CXCR2-Dependent Pathway

**Figure 5:**
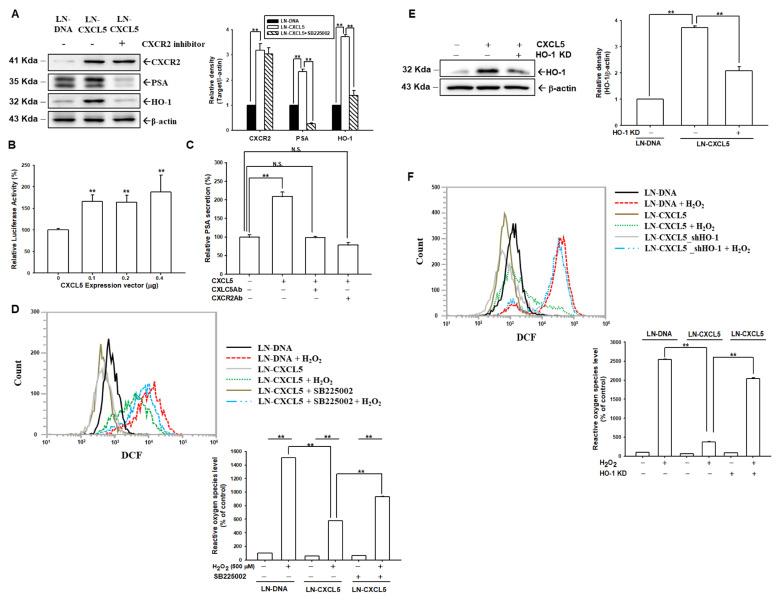
Modulation of CXCL5 via CXC2R in endogenous and H_2_O_2_-induced ROS in prostate cancer LNCaP cells. (**A**) The protein levels of CXCR2, PSA, and HO-1 of LN-DNA, LN-CXCL5, and SB225002-treated LN-CXCL5 cells were examined by immunoblot assays. Quantitative analysis (±SE, *n* = 3) is presented as the relative density of target proteins/β-actin. (**B**) Reporter activity (±SE, *n* = 6) of the PSA reporter vector after co-transfected with various doses of CXCL5 expression vectors. (**C**) PSA levels (±SE, *n* = 6) in the supernatant of LN-DNA, LN-CXCL5, and LN-CXC5 treated with CXCL5 antibody or CXCR2 antibody, as assessed by ELISA. Data are presented as PSA secretion in relation to the LN-DNA group. (**D**) ROS levels and quantitative data (±SE, *n* = 3) of LN-DNA and LN-CXCL5 cells after treatment with or without H_2_O_2_ and SB225002, as measured by flow cytometry. (**E**) HO-1 protein levels when LN-CXCL5 cells were transiently knocked down as to the HO-1 gene by immunoblot assays and quantitative analysis (±SE, *n* = 3). (**F**) ROS levels and quantitative data (±SE, *n* = 3) from LN-DNA, LN-CXCL5, and HO-1-knockdown LN-CXCL5 cells after treatment with/without H_2_O_2_, as measured by flow cytometry. ** *p* < 0.01. N.S., no significant difference.

Immunoblot assays and quantitative analysis showed that CXCL5 overexpression enhanced CXCR2, PSA, and HO-1 expressions in LNCaP cells (Figure 5A). However, the increasing activity of CXCL5 on these proteins was blocked when co-treated with SB225002. The reporter assays revealed that transient CXCL5-overexpressed LNCaP cells induced the activity of the PSA reporter (Figure 5B). The results of ELISA revealed that the overexpression of CXCL5 increased PSA secretion compared to LN-DNA cells; however, the activation of CXCL5 was blocked when cotreated with CXCL5 or CXCR2 antibodies (Figure 5C).

### 3.7. CXCL5 Overexpression Blocks H_2_O_2_-Induced ROS in LNCaP Cells by Inducing HO-1

Immunoblot assays found that CXCL5 upregulated HO-1 expression, suggesting that CXCL5 might have antioxidant characteristics. The flow cytometry assays and the quantitative analysis indicated that the 500 μM H_2_O_2_ treatment induced a 15.05-fold increase in ROS in LN-DNA cells. However, the same concentration of H_2_O_2_ treatment only induced a 5.76-fold increase in ROS in LN-CXCL5 cells. Further investigation illustrated that the blocking effect of CXCL5 on H_2_O_2_-induced ROS was attenuated by cotreatment with SB225002 (Figure 5D), indicating that CXCL5 overexpression affects endogenous and H_2_O_2_-induced ROS in LNCaP cells that are CXCR2-dependent. To determine the function of HO-1, as regulated by CXCL5 in cellular ROS stress, we cloned CXCL5-overexpressed-with-HO-1-knockdown LNCaP cells. Immunoblot assays and quantitative analysis showed that CXCL5 overexpression upregulated HO-1 expression, but HO-1 activation by CXCL5 was blocked by HO-1 knockdown in LNCaP cells (Figure 5E). The flow cytometry assays and quantitative analysis indicated similar results, in which the overexpression of CXCL5 blocked H_2_O_2_-induced ROS levels in LNCaP cells. However, the decreasing effect of CXCL5 on ROS was reversed when the HO-1 gene was knocked down in LN-CXCL5 cells (Figure 5F), suggesting that CXCL5 blocked H_2_O_2_-induced ROS dependent on upregulation of the expression of the HO-1 gene.

### 3.8. Functions of CXCL5 on Human Myofibroblast WPMY-1 Cells

**Figure 6:**
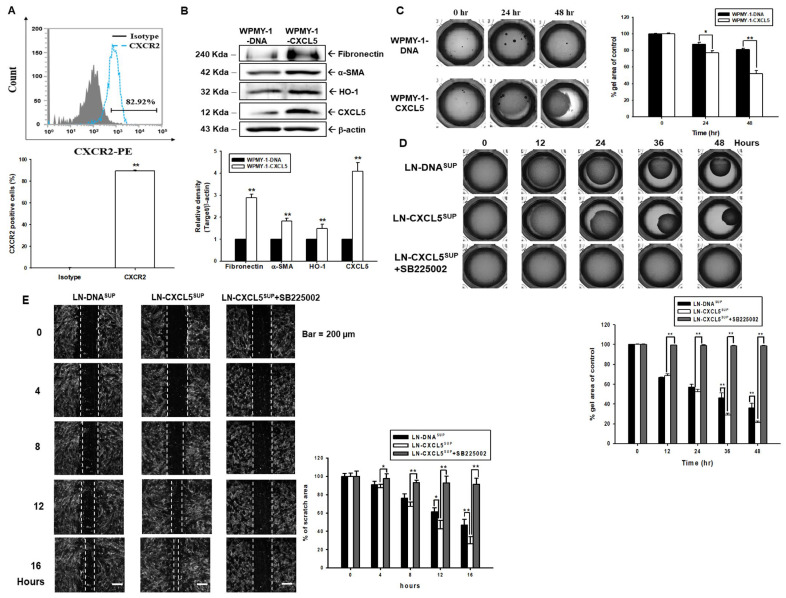
Modulation of CXCL5 in cell contraction and migration in prostate stroma myofibroblast WPMY-1 cells. (**A**) Cell immunofluorescent staining of the CXCR2 protein in WPMY-1 cells was determined by flow cytometry. The data from the quantitative analysis represents the percentage of CXCR2-positive cells. (**B**) Protein levels of Fibronectin, α-SMA, HO-1, CXCL5, and β-actin in WPMY-1-DNA and WPMY-1-CXCL5 cells, as determined by immunoblot assays and quantitative analysis (±SE, *n* = 3). (**C**) Cell contraction of mock-transfected WPMY-1 (WPMY-1-DNA) and CXCL5-overexpressed WPMY-1 (WPMY-1-CXCL5) cells, as measured by collagen contraction assays. Data are presented as the mean percentage (±SE; *n* = 3) of WPMY-1-CXCL5 cells in relation to WPMY-1-DNA cells. (**D**) Cell contraction of WPMY-1 cells when treated with the supernatant from the LN-DNA, LN-CXCL5, or LN-CXCL5 with SB225002. Data are presented as the mean percentage (±SE; *n* = 3) in relation to the supernatant from LN-DNA-treated WPMY-1 cells. (**E**) Migration capabilities in WPMY1 cells treated with conditioned media of LN-DNA, LN-CXCL5, or LN-CXCL5 with SB225002. The white line indicates the average of the leading edges of cells and the size of the wound area. Data are presented as the mean percentage (±SE; *n* = 3) in relation to the supernatant from LN-DNA-treated WPMY-1 cells. * *p* < 0.05, ** *p* < 0.01.

As shown in Figure 1, human myofibroblast WPMY-1 cells also express CXCL5, although none of the reports have discovered its function. The results of cell immunofluorescent staining determined by flow cytometry analysis showed that WPMY-1 cells express CXCR2 (Figure 6A). Immunoblot assays and quantitative analysis showed that transient overexpression of CXCL5 enhanced the expressions of CXCL5, HO-1, alpha-smooth muscle actin (α-SMA), and fibronectin (Figure 6B). Collagen-based cell contraction assays and quantitative analysis demonstrated that overexpression of CXCL5 in WPMY-1 cells (WPMY-1-CXCL5) enhanced the cell contraction compared to WPMY-1 cells that were mock-transfect (WPMY-1-DNA) (Figure 6C). Similar results were also found when WPMY-1 cells treated with LN-DNA cell supernatant had less contraction ability than those cultured with LN-CXCL5. Furthermore, the contraction was blocked when WPMY-1 cells were cotreated with 2 μM SB225002 (Figure 6D). Wound healing assays and quantitative analysis showed similar results, indicating that the LN-CXCL5 cell supernatant promoted WPMY-1 cell migration, which is CXCR2-dependent (Figure 6E).

### 3.9. CXCL5 Blocks H_2_O_2_-Induced ROS in WPMY-1 Cells

**Figure 7:**
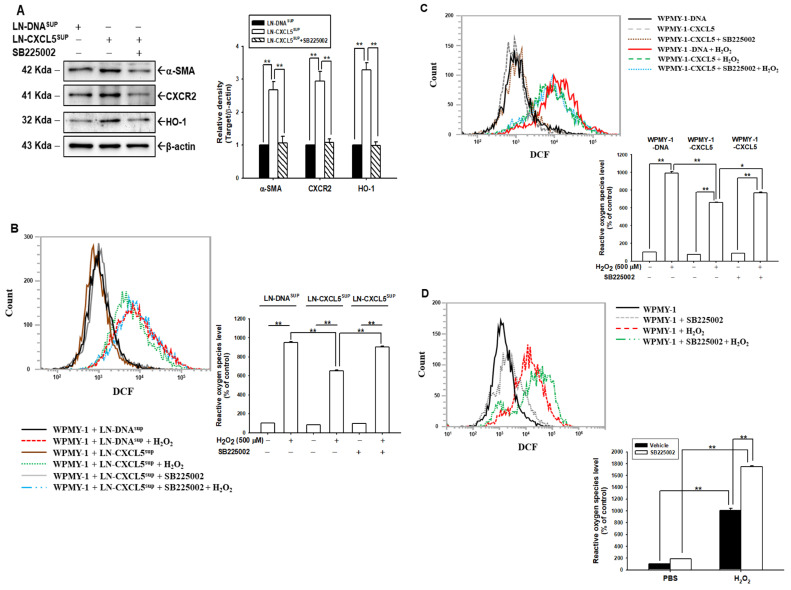
Modulation of CXCL5 and SB225002 in endogenous and H_2_O_2_-induced ROS in prostate stroma myofibroblast WPMY-1 cells. (**A**) The protein levels of α-SMA, CXCR2, and HO-1 of WPMY-1 cells after treatment with the conditioned media of the LN-DNA, LN-CXCL5, or LN-CXCL5 with SB225002, as examined by immunoblot assays. Quantitative analysis (±SE, *n* = 3) is presented as the relative density of target proteins/β-actin. (**B**) ROS levels and quantitative data for WPMY-1 cells after treatment with the conditioned media of the LN-DNA, LN-CXCL5, or LN-CXCL5 with SB225002, and with/without H2O2, as measured by flow cytometry. (**C**) ROS levels and quantitative data from WPMY-1-DNA, WPMY-1-CXCL5, and SB225002-treated WPMY-1-CXCL5 cells, after treatment with or without H_2_O_2_, as measured by flow cytometry. (**D**) ROS levels and quantitative data for WPMY-1 cells after treatment with 2 μM SB225002 and with/without H_2_O_2_, as measured by flow cytometry. * *p* < 0.05, ** *p* < 0.01.

Immunoblot assays and quantitative analysis showed that WPMY-1 cells cultured with LN-CXCL5 cell supernatant expressed higher protein levels of α-SMA, CXCR2, and HO-1 in WPMY-1 cells than in cells cultured with the LN-DNA cell supernatant. The cotreatment with SB225002 blocked the expressions of α-SMA, CXCR2, and HO-1, which had been induced by the supernatant from LN-CXCL5 cells (Figure 7A). The flow cytometry assays and quantitative analysis indicated ROS levels of WPMY-1 cells after treatment with the conditioned media from the LN-DNA and LN-CXCL5 cells with H_2_O_2_ treatment. However, the supernatant from LN-CXCL5 treatment more strongly attenuated H_2_O_2_-induced ROS levels, compared to the LN-DNA treatment. This attenuating effect of LN-CXCL5 supernatant treatment was reversed when WPMY-1 cells were co-treated with LN-CXCL5 supernatant and SB225002. These results indicated that the supernatant from LN-CXCL5 cells induced expression of HO-1, which reduced the levels of ROS induced by H_2_O_2_ in WPMY-1 cells, while SB225002 inhibited these effects, suggesting that CXCL5 secretion from prostate cancer cells enhanced the antioxidation of WPMY-1 cells (Figure 7B). The results shown in Figure 6C reveal that transient overexpression of CXCL5 enhanced the expression of HO-1; we then continued on to determine whether CXCL5 also blocked endogenous and H_2_O_2_-induced ROS levels in WPMY-1 cells. Flow cytometry assays showed that transient overexpression of CXCL5 decreased endogenous ROS and H_2_O_2_-induced ROS in WPMY-1 cells (Figure 7C). As mentioned above, WPMY-1 cells expressed detectable levels of CXCL5; we also determined whether SB225002 affected endogenous ROS and H_2_O_2_-induced ROS. The flow cytometry assays and quantitative analysis indicated that 500 μM H_2_O_2_ treatment increased ROS levels in WPMY-1 cells with or without SB225002 treatment. However, SB225002 treatment enhanced endogenous ROS and H_2_O_2_-induced ROS in WPMY-1 cells (Figure 7D). Collectively, these results illustrate that CXCL5 functions as the crosstalk between stroma and cancer cells, and CXCL5 as an antioxidant depends on CXCR2 in both WPMY-1 and LNCaP cells.

## 4. Discussion

CXCL5 is a proangiogenic CXC-type chemokine that is an inflammatory mediator; it is secreted not only by immune cells but also by epithelial cells, endothelial cells, and fibroblasts [8]. Early studies identified that serum CXCL5 levels were the relevant marker for men with prostate diseases, including benign prostatic hyperplasia, prostatitis, and cancer progression [3,4,5,6,9,32]. After specific binding to its receptor, CXCR2 (C-X-C chemokine receptor type 2), a member of the G protein-coupled receptor (GPCR) family, CXCL5 is chemotactic for a series of biological effects, such as neutrophil migration [8,17,33]. This study illustrated that the supernatant from LNCaP overexpressed with CXCL5 enhanced neutrophil migration compared to the supernatant of mock-transfected LNCaP cells. In contrast, the supernatant of PC-3 cells knocked down with CXCL5 presented a weaker chemotactic effect than mock-transduced PC-3 cells, indicating that CXCL5 secreted from LNCaP cells overexpressed with CXCL5 and endogenous CXCL5 from PC-3 cells have the biological activity of CXCL5, and attract neutrophil migration. The chemotactic characteristics of CXCL5, which induce neutrophil migration to affect tumorigenesis, are well-known in several cancers, including prostate cancer [8,9,34,35,36]. Our study also confirmed that CXCR2 is expressed in LNCaP and PC-3 cells, a finding consistent with those of other studies [18,37]. A previous study indicated that PTEN blocked the CXCL5 expression in renal cell carcinoma [38]. That may explain why DU145 cells, an aggressive cell line, expressed extremely low levels of CXCL5 in this study; namely, DU145 cells are PTEN-positive prostate cancer cells [39]. Further investigation of PTEN on CXCL5 expression in prostate cancer cells is warranted.

The results of the present study showed that CXCL5 enhanced cancer cell growth in vitro and in xenograft animal studies, a finding which agrees with other studies that have found CXCL5 to be associated with tumor progression in prostate cancer [6,7,40]. The results of the Ki67 proliferation assay showed that knockdown CXCL5 attenuated cell proliferation of PC-3 cells, although a previous study indicated that exogenously administrated CXCL5 did not affect the cell proliferation of PC-3 cells in vitro [6]. These divergent results may be due to high levels of endogenous CXCL5 in the PC-3 cells. This study showed that CXCL5 induced PC-3 cell invasion through epithelial–mesenchymal transition (EMT) modulation (Figure 1), which is consistent with an earlier study [40]. Furthermore, CXCL5 modulated the proteins of cell cycle regulators, which promote cyclins and cyclin-dependent kinases (CDK), and downregulated CDK inhibitors to enhance cell cycle S phase and downregulation of the G1 phase, a finding which is supported by another study [41].

Recent studies have underscored the crucial role of CXCR2 in prostate cancer metastasis, particularly as to bone, a common site for prostate cancer spread. The influence of CXCR2 and its ligands on the interaction between cancer cells and the bone microenvironment supports the growth and survival of metastatic lesions. In particular, prostate-specific antigen (PSA) and CXCL5 levels are known to increase in the serum of prostate cancer patients [10,42]. However, there was no direct evidence to support that PSA levels were correlated with CXCL5 in the tissues or sera of clinical patients, which may be due to the divergent multi-tumor microenvironments that modulate them. The results of the present study have revealed that CXCL5 upregulated the expression of PSA in LNCaP cells in vitro. Importantly, we found that CXCL5 treatment-induced PSA secretion was blocked by co-treating with the CXCL5 antibody and the CXCR2 antibody, indicating that CXCL5 upregulated PSA gene expression through the CXCL5/CXCR2 axis (Figure 5). It had been known that CXCL5 mediated the NF-κB pathway, a key signaling pathway in cancer, and the PSA is the target gene of NF-κB [43,44]. The precise mechanisms, including the NF-κB signaling pathway, of CXCL5-regulated PSA expression warrant further investigation.

HO-1 has anti-inflammatory, antiapoptotic, and antioxidant properties in cancer cells and is deemed a tissue protector functioning to promote tumor growth [26,45,46]. A previous study revealed that HO-1 attenuated ROS induced by H_2_O_2_ treatment in PC-3 and DU145 cells [26]. To date, no reports have indicated the regulation of CXCL5 in the HO-1 gene, although a recent study illustrated the antioxidant effect of CXCL5 in white adipocytes [11]. However, another study showed that CXCL5 induced mitochondrial ROS in cholangiocarcinoma cells [47]. The present study is the first to determine the effect of CXCL5 on the expression of the HO-1 gene and its modulation of ROS in prostate cancer and myofibroblast cells. These studies illustrated that CXCL5, through the expression of HO-1, affects endogenous ROS and blocks H_2_O_2_-induced ROS levels in prostate cancer cells. HO-1 attenuated ROS in prostate cancer cells, a finding consistent with previous studies [26]. Furthermore, treatment with the CXCR2 inhibitor SB225002 blocked HO-1 expression and upregulated endogenous ROS and H_2_O_2_-induced ROS levels in prostate cancer cells (Figure 3 and Figure 5), indicating that CXCL5 affects endogenous ROS and H_2_O_2_-induced ROS, and is dependent on HO-1 and CXCR2.

CXCL5 is known to be secreted by stroma and cancer cells and via autocrine and paracrine pathways to modulate tumorigenesis in prostate and liver cancers [21,48]; however, only a few reports have discovered the function of CXCL5 in prostate stroma cells. A recent study revealed that stroma CXCL5 is involved in prostate cancer progression and metastasis [49]. The present study showed that prostate myofibroblast WPMY-1 cells express detectable levels of CXCL5, a finding consistent with previous studies [7,49]. Collagen-based cell contraction and migration assays showed that CXCL5 enhanced the contraction and migration abilities of WPMY-1 cells; moreover, SB225002, a CXCR2 antagonist, blocked the activation of CXCL5 in the contraction and migration of WPMY-1 cells. The increased cell migration and contraction of CXCL5 may be due to the upregulation of fibronectin and α-SMA, two proteins involved in cell motility [50,51]. CXCL5-induced fibronectin expression is consistent with a previous study on circulating tumor cells [52]. Recent studies indicated that several CXCR2 antagonists have promise for the reduction of tumor growth and metastasis in prostate cancer [14]. This study is the first to illustrate the effect of CXCR2 antagonists on the contraction and migration of prostate stroma cells.

## 5. Conclusions

The antioxidant functions of CXCL5 in the human prostate remain unclear, although it is a well-known attractant of immune cells and an inflammatory mediator. CXCL5 secreted from prostate cancer cells enhanced neutrophil migration, indicating the chemotactic characteristics of CXCL5 in prostate cancer cells. CXCL5 modulates the cell cycle and EMT to induce cell proliferation and invasion of prostate cancer cells in vitro and tumor growth in xenograft animal models. Furthermore, CXCL5 enhanced the migration and contraction of WPMY-1 cells. Overexpression of CXCL5 blocked H_2_O_2_-induced ROS in prostate myofibroblasts and cancer cells. CXCL5 activation in antioxidants is reversed by removing CXCL5 or HO-1 or pre-retreating with a CXCR2 inhibitor. This study explored the concept that CXCL5 is an antioxidant oncogene through the CXCL5/CXCR2/HO-1 pathway in prostate cancer cells, suggesting that the CXCL5/CXCR2/HO-1 signaling pathway could provide a new strategy for the treatment of oxidative stress in the prostate.

## Data Availability

All of the data are presented in this article.
